# Novel diagnostic approach on the identification of *Brucella melitensis* Greek endemic strains‐discrimination from the vaccine strain Rev.1 by PCR‐RFLP assay

**DOI:** 10.1002/vms3.99

**Published:** 2018-03-22

**Authors:** Sofia Christoforidou, Evridiki Boukouvala, Antonios Zdragas, Eleni Malissiova, Vassilios Sandalakis, Anna Psaroulaki, Evanthia Petridou, Panagiotis Tsakos, Loukia Ekateriniadou, Christos Hadjichristodoulou

**Affiliations:** ^1^ Veterinary Research Institute of Thessaloniki Hellenic Agricultural Organization DEMETER (former NAGREF) Thessaloniki Greece; ^2^ Dairy Laboratory Food Technology Department Technological Educational Institute of Thessaly Thessaly Greece; ^3^ Laboratory of Clinical Bacteriology, Parasitology, Zoonoses and Geographical Medicine School of Medicine University of Crete Heraklion Greece; ^4^ Laboratory of Microbiology Faculty of Veterinary Medicine Aristotle University of Thessaloniki Thessaloniki Greece; ^5^ Ministry of Rural Development and Food Directorate of Veterinary Centre of Thessaloniki Laboratory of Microbiology & Infectious Diseases Thessaloniki Greece; ^6^ Laboratory of Hygiene and Epidemiology Faculty of Medicine School of Health Sciences University of Thessaly Larissa Greece

**Keywords:** biotyping, *Brucella melitensis*, multiplex PCR, real‐time PCR, RFLP

## Abstract

Despite the intensive implementation of control programmes goat, sheep and human brucellosis remains endemic in Greece. As the discrimination between field endemic strains and vaccine strain Rev.1 is not feasible, it is essential to develop new diagnostic tools for brucellosis diagnosis. Moreover, effective disease control requires enhanced epidemiological surveillance in both humans and animals including robust laboratory support. Two new multiplex (duplex) polymerase chain reactions (PCRs) were developed and the results were compared with those obtained by real‐time PCR and bacteriological biotyping. A total of 71 *Brucella* spp. Greek endemic strains were identified at species and biovar level, using both molecular and conventional techniques. Their discrimination from the vaccine strain Rev.1 was achieved, using polymerase chain reaction‐restriction fragment length polymorphism assay (PCR‐RFLP). All 71 strains were identified as *Brucella melitensis* by multiplex PCR as well as by real‐time PCR and conventional biotyping. Sixty‐two (87.3%) out of 71 strains were identified as *B. melitensis* biovar 3, eight (11,3%) strains as biovar 1 and only one (1,4%) as biovar 2. Digestion with *Pst*I restriction enzyme revealed that all strains were field endemic strains, as they gave different patterns from the vaccine strain Rev.1. *Brucella melitensis* biovar 3 appears to be the predominant type in Greece. The novel multiplex PCR produced results concordant to ones obtained by real‐time PCR and conventional biotyping. This technique could support and facilitate the surveillance of Brucellosis in Greece contributing in the control of the disease.

## Introduction

Brucellosis is a zoonotic disease caused by Gram‐negative, facultative, intracellular bacteria (Alton *et al*. 1975). Within the genus *Brucella,* six species have been described according to their phenotypic characteristics, antigenic variation and preferential host: *B. melitensis, B. abortus, B.suis, B. canis, B. ovis*, and *B. neotomae* (Moreno *et al*. 2002). Recently, four new species have been recognized: *B. ceti, B. pinnipedialis* (Foster *et al*. 2007)*, B. microti* (Scholz *et al*. 2008a, 2009), and *B. inopinata* (Scholz *et al*. 2010).

Although there was a significant decreasing trend in reported numbers of human brucellosis in Europe between 2006 and 2009, the disease is still prevalent in southern European countries such as Greece, Spain, Portugal (ECDC, 2011), and southern Italy (Campania, Apulia, Calabria, Sicily) (Mancini *et al*. 2014). According to the World Health Organization (WHO), the incidence of brucellosis worldwide and especially in developing countries is estimated to be 10–25 times higher than the recorded due to under‐reporting (FAO/WHO, 1986).

The majority of reported cases are attributed to infections by *B. melitensis, B. abortus* and *B. suis*, in descending order of prevalence (Al Dahouk *et al*. 2013). Of these three species, infections by *B. melitensis* are the most common and most serious in humans (Pappas *et al*. 2005). People contract the disease either by direct contact with animals and/or their secretions, or by consuming contaminated unpasteurized milk and dairy products (Díaz Aparicio 2013).

*Brucella melitensis* has three biovars (1, 2 and 3). All three biovars cause disease in small ruminants, but their geographic distribution varies. Biovar 3 is predominant in the Mediterranean countries and the Middle East (Samadi *et al*. 2010), while biovar 1 predominates in Central and South America (Gaido *et al*. 2011) but has also been reported in some Mediterranean countries such as Turkey (Erdenlig *et al*. 2011) and Spain (Díaz Aparicio *et al*. 1994). In Greece, to the best of our knowledge, there is a lack of data concerning the predominance of specific *B. melitensis* biovars.

As ovine and caprine brucellosis, due to *B. melitensis*, remains a significant problem for both public health and animal husbandry in Greece (ECDC, 2011), the State Veterinary Services of the Ministry of Rural Development and Food have implemented a control and eradication strategy to decrease economic losses, based on systematic vaccination on the mainland, and a test and slaughter policy on the islands, excluding the islands of Lesvos, Leros, Thassos and Evia (HCDCP, 2012). The vaccination of small ruminants against the disease is based on the conjunctival administration of the attenuated live vaccine strain, Rev.1. However, retrospective data have shown the inefficacy of the vaccination programme as the administration of the Rev.1 vaccine can lead to strain persistence in the vaccinated animals, may cause human infection and can be spread horizontally (Banai *et al*. 1996; Saaedzadeh *et al*. 2013). In order to overcome the aforementioned problems, researchers (Bardenstein *et al*. 2002) suggest that a polymerase chain reaction‐restriction fragment length polymorphism (PCR‐RFLP) assay could be used to differentiate vaccine strain Rev.1 from field strain infection by studying the polymorphisms of *omp2* gene (*omp2a* and *omp2b* alleles*)*, using *Pst*I endonuclease enzyme.

Several identification methods have been used to ensure effective brucellosis disease prevention and control. At present, the most common methods for the diagnosis of brucellosis are the isolation of the causative agent by culture, the serological tests and the molecular techniques (Nielsen & Yu 2010; Al Dahouk & Nöckler 2011). The bacterial isolation, despite its drawbacks (time‐consuming, laboratory risk infection, dependence on the viability, and numbers of *Brucella* spp. in the sample), is still the “gold standard” method for brucellosis diagnosis (Refai 2003). Serological tests seem to be more effective, but problems may arise concerning cross‐reacting antibodies and the lack of variable cut‐offs for different levels of endemicity (Al Dahouk *et al*. 2013). Recently, studies have shown that fluorescent polarization assay (FPA) is a promising method for replacing other serological tests for human brucellosis diagnosis due to its high speed, low cost, and the objectivity of results interpretation. However, further studies are needed to assess the reproducibility of FPA (Konstantinidis *et al*. 2007).

As none of these two diagnostic tools can be used on its own to detect the causative agent reliably, various molecular techniques (PCR, real‐time PCR, sequencing) have been developed to overcome the difficulties of brucellosis diagnosis. These techniques have shown promising results regarding their sensitivity and specificity, while at the same time are easy to perform, avoid the risk of laboratory infection, and require a short period of time (Surucuoglu *et al*. 2009).

The aims of this study were as follows: (1) the development of new multiplex PCR methods to identify strains at genus and species level, (2) the comparative evaluation of the new multiplex PCR method with a real‐time PCR assay, and the bacteriological biotyping, (3) the sub‐typing of *B. melitensis* strains for the epidemiological surveillance of the disease in Greece, as there is a lack of data concerning the endemic field strains and (d) the discrimination of the vaccine strain Rev.1 from the endemic *B. melitensis* strains by PCR‐RFLP.

## Materials and methods

### Collection and description of bacterial strains

A total of 71 *Brucella* strains endemic in Greece were collected between 1999 and 2010. Twenty‐nine *Brucella* strains of goat and sheep origin were isolated from liver, spleen tissues, lymph nodes and aborted fetuses, whereas 42 human isolates were from blood. All human strains originated from the island of Crete (Medical School of University of Crete), whereas strains of animal origin came from the regions of Macedonia and Thessaly in northern (Centre of Veterinary Institutes of Thessaloniki) and central Greece (Veterinary School of Aristotle University of Thessaloniki), respectively (Table 1). The analyses were performed in the Veterinary Research Institute of Thessaloniki, Greece.

**Table 1:** Epidemiological data of strains analysed in this study

No of *B. melitensis* strains	Strains source	Strains origin
10	Goat and sheep tissues	Region of Macedonia
19	Goat and Sheep tissues	Region of Thessaly
42	Human blood	Region of Crete

### Identification of strains by bacteriological characteristics

Strains were cultured on plates of tryptic soy agar supplemented with 5% equine serum, 1–5% dextrose 25% and six antibiotics. The following concentrations of antibiotics were added per litre of media: polymyxin B sulphate (5.000 units), bacitracin (25.000 units), natamycin (50 mg), nalidixic acid (5 mg), nystatin (100.000 units), and vancomycin (20 mg)^1^. The plates were incubated at 37°C for 48–72 h. Biotyping of *B. melitensis* strains was achieved, using tests based on growth characteristics, requirement of added carbon dioxide, dye tolerance, production of hydrogen sulfide and agglutination in monospecific anti‐sera A and M (Alton *et al*. 1975). In all the aforementioned bacteriological methods, the vaccine strain Rev.1 and the reference strain 16M were used as controls.

### Extraction of Brucella genomic DNA

The extraction of genomic DNA was performed with the use of PureLink Genomic DNA kit^2^ for Gram‐negative bacteria. The whole process was performed in P3 conditions to avoid laboratory contamination.

### Development of a new multiplex PCR for the identification at genus and species level

Several primers were examined based on previous studies, ultimately, using the ones described by Scholz *et al*. 2008b and Imaoka *et al*. 2007;. For the development and validity of multiplex PCR, five control strains were used; one *B. melitensis*, one *B. abortus,* one *B. suis*, one *B. ovis* and one *B. canis,* kindly offered by Istituto Zooprofilattico Sperimentale dell' Abruzzo e Molise in Teramo, Italy and five non‐related pathogenic bacteria such as *Staphylococcus aureus, Bacillus megaterium, Salmonella typhimurium, Shigella flexneri* and *Escherichia coli* 8879, kindly provided by the School of Biology of Aristotle University of Thessaloniki. Finally multiplex PCRs were performed for the detection of *Brucella* spp.*, B. melitensis, B. abortus, B. suis, B. ovis* and *B. canis*. Multiplex PCR consisted of two duplex PCRs, one based on the use of two primers pairs (B4/B5 and BrucR/F), responsible for the identification of strains at genus level (Scholz *et al*. 2008b) and one based on the use of primers pairs JPF‐F/JPF‐abR and 1S(F)/1AS(R), responsible for the identification of *B. melitensis, B. abortus, B. suis*,* B. ovis* and *B. canis*. (Imaoka *et al*. 2007). The sequences of the oligonucleotide primers are presented in Table 2.

**Table 2:** Oligonucleotide primers used in the multiplex PCR assay for the detection of *Brucella* spp., *B. abortus*,* B. melitensis*,* B. suis, B. ovis* and *B. canis* (Imaoka *et al*. 2007; Scholz *et al*. 2008b)

Primer name	Sequence (5′‐3′ orientation)	Target gene	Amplicon (bp)
Bruc‐F	5′‐AACCACGCTTGCCTTGCACACC‐3′	*recA*	167
Bruc‐R	5′‐TTTCAAGCGCCTGTTCACCCCG ‐3′		
B4	5′‐TGGCTCGGTTGCCAATATCAA‐3′	*bcsp31*	223
B5	5′‐CGCGCTTGCCTTTCAGGTCTG‐3′		
JPF‐F	5′‐GCGCTCAGGCTGCCGACGCAA‐3′	*omp2*	186
JPF‐abR	5′‐CATTGCGGTCGGTACCGGAG‐3′		
1S‐F	5′‐GTTCGCTCGACGTAACAGCTG‐3′	*omp31*	249
1AS‐R	5′‐GACCGCCGGTACCATAAACCA‐3′		

The PCR reactions were performed in a 2720 Thermal Cycler^3^ in 10 *μ*L final volume. Each reaction consisted of 1 × KAPA 2G Multiplex PCR kit mix, 300 nmol/L of each primer and 50–70 ng genomic DNA. The PCR started with an initial denaturation step at 95°C for 3 min, followed by 30 cycles of denaturation at 95°C for 15 s, annealing at 60°C for 30 s, extension at 72°C for 20 s and final extension at 72°C for 3 min. The PCR products underwent electrophoresis on agarose gel 1.5% in 0.5X TBE buffer and were visualized by staining with ethidium bromide (15 *μ*g).

### Identification at genus and species level by real‐time PCR analysis

The real‐time PCR in our study was mainly performed as to confirm the results of the multiplex PCR and the bacteriological examination. Two duplex qPCRs were performed, one for the identification of *B. *spp. and *B. melitensis* and one for the identification of *B. *spp. and *B. abortus*. As target genes for the identification of *B. *spp*., B. melitensis* and *B. abortus* were used, the multiple insertion element IS711, the BMEII0466 and BruAb2_0168 genes, respectively. The primer pairs and probes that were used are described previously (Hinić *et al*. 2008) and are presented in Table 3. The reaction mixtures were prepared at a final volume of 25 *μ*L, containing 12.5 *μ*L of Platinum Quantitative qPCRSupermix‐UDG^4^, 300 nmol/L of each primer, 250 nmol/L of each probe and 60–80 ng DNA. The probe for the identification of *B. *spp. was labelled with Cy5, for *B. melitensis* with VIC and for *B. abortus* with FAM. Real‐time qPCRs were performed in a Chromo4^TM^RealTime Detector. The cycling conditions for all the reactions consisted of an initial denaturation at 95°C for 3 min, and finally 45 cycles of denaturation at 95^°^C for 15 s and annealing/extension at 60°C for 30 s.

**Table 3:** Real‐time PCR primer sequence for the detection and species identification of *Brucella* spp (Hinić *et al*. 2008)

PCR	Target sequence	Forward primer/reverse primer (5′→3′)	Probe (5′Fluorophore→3′Quencher)
1	IS711	GCTTGAAGCTTGCGGACAGT/GGCCTACCGCTGCGAAT	Cy5‐AAGCCAACACCCGGCCATTATGG‐BHQ2
2	BMEII0466	TCGCATCGGCAGTTTCAA/CCAGCTTTTGGCCTTTTCC	VIC‐CCTCGGCATGGCCCGCAA‐BHQ1
3	BruAb2_0168	GCACACTCACCTTCCACAACAA/CCCCGTTCTGCACCAGACT	FAM‐TGGAACGACCTTTGCAGGCGAGATC‐BHQ1

### Discrimination from vaccine strain Rev.1 by the analysis of omp2 gene by PCR‐RFLP

For the discrimination of field strains from the vaccine strain Rev.1, PCR‐RFLP assay was performed as described in previous studies (Bardenstein *et al*. 2002; Noutsios *et al*. 2012). The differentiation was achieved by studying the polymorphisms of the *omp2* gene (*omp2a* and *omp2b* alleles). The primers that were used have been described previously (Bardenstein *et al*. 2002). Amplification reaction mixtures were prepared in a total volume of 25 *μ*L containing 1x PCR ThermoPol II Buffer^4^, 5 mmol/L MgCl_2_, 0.8 mmol/L dNTPs, 1 *μ*mol/L of each primer, 80–100 ng of genomic DNA and 1.25 U Taq recombinant polymerase^4^. The temperature cycling for amplification was performed in a 2720 Thermal Cycler^5^ as follows: initial denaturation step at 94°C for 2 min, followed by 35 cycles of denaturation at 94°C for 20 s, annealing at 60°C for 1 min, extension at 72°C for 1 min and final extension at 72°C for 7 min. Digestion of the amplified omp2 genes was performed with *Pst*I restriction enzyme^6^ at 37°C for 2 h. The digested DNA was separated by electrophoresis on agarose gel 3% in 0.5X TBE buffer. DNA fragments were visualized by staining with ethidium (1.5 *μ*g mL/L).

## Results

### Identification of strains by bacteriological characteristics

Colony and cellular morphology as well as the results of biochemical tests were compatible with those described for the genus *Brucella*. The growth pattern on basic fuchsin and thionin were consistent with those for *B. melitensis*, as all strains grew on basic fuchsin 1:50.000 and 1:100.000 and thionin 1:50.000 and 1:100.000, whereas no culture development was observed on thionin 1:25.000. Moreover, the same growth was observed for all strains either in aerobic conditions or in the presence of added carbon dioxide, but no hydrogen sulfide production was present during the 4 days of cultivation. Agglutination with monospecific antisera A & M revealed that 62 out of 71 strains (87.3%) were identified as *B. melitensis* biovar 3, one strain (1.4%) was *B. melitensis* biovar 2 and eight strains (11.3%) were identified as *B. melitensis* biovar 1. The results are demonstrated analytically in Table 4. The 62 strains that were identified as *B. melitensis* biovar 3 originated from all three regions of Greece, whereas *B. melitensis* biovar 1 was found mainly in northern (three strains) and central Greece (five strains) and *B. melitensis* biovar 2 in Thessaloniki, northern Greece.

**Table 4:** Identification of field strains by bacteriological characteristics

No of Strains	CO_2_ requirement	H_2_S production	Sensitivity to dyes					Serum agglutination		Identification results
			Basic fuchsin		Thionin					
			1:50.000	1:100.000	1:25.000	1:50.000	1:100.000	A	M	
1	+/−	−	+	+	−	+	+	+	+	*B. melitensis* bv.3
2	+/−	−	+	+	−	+	+	+	+	*B. melitensis* bv.3
3	+/−	−	+	+	−	+	+	+	+	*B. melitensis* bv.3
4	+/−	−	+	+	−	+	+	+	+	*B. melitensis* bv.3
5	+/−	−	+	+	−	+	+	+	+	*B. melitensis* bv.3
6	+/−	−	+	+	−	+	+	+	+	*B. melitensis* bv.3
7	+/−	−	+	+	−	+	+	+	+	*B. melitensis* bv.3
8	+/−	−	+	+	−	+	+	+	+	*B. melitensis* bv.3
9	+/−	−	+	+	−	+	+	+	+	*B. melitensis* bv.3
10	+/−	−	+	+	−	+	+	+	+	*B. melitensis* bv.3
11	+/−	−	+	+	−	+	+	+	+	*B. melitensis* bv.3
12	+/−	−	+	+	−	+	+	+	+	*B. melitensis* bv.3
13	+/−	−	+	+	−	+	+	+	+	*B. melitensis* bv.3
14	+/−	−	+	+	−	+	+	+	+	*B. melitensis* bv.3
15	+/−	−	+	+	−	+	+	+	+	*B. melitensis* bv.3
16	+/−	−	+	+	−	+	+	+	+	*B. melitensis* bv.3
17	+/−	−	+	+	−	+	+	+	+	*B. melitensis* bv.3
18	+/−	−	+	+	−	+	+	+	+	*B. melitensis* bv.3
19	+/−	−	+	+	−	+	+	+	+	*B. melitensis* bv.3
20	+/−	−	+	+	−	+	+	+	+	*B. melitensis* bv.3
21	+/−	−	+	+	−	+	+	+	+	*B. melitensis* bv.3
22	+/−	−	+	+	−	+	+	+	+	*B. melitensis* bv.3
23	+/−	−	+	+	−	+	+	+	+	*B. melitensis* bv.3
24	+/−	−	+	+	−	+	+	+	+	*B. melitensis* bv.3
25	+/−	−	+	+	−	+	+	+	+	*B. melitensis* bv.3
26	+/−	−	+	+	−	+	+	+	+	*B. melitensis* bv.3
27	+/−	−	+	+	−	+	+	+	+	*B. melitensis* bv.3
28	+/−	−	+	+	−	+	+	+	+	*B. melitensis* bv.3
29	+/−	−	+	+	−	+	+	+	+	*B. melitensis* bv.3
30	+/−	−	+	+	−	+	+	+	+	*B. melitensis* bv.3
31	+/−	−	+	+	−	+	+	+	+	*B. melitensis* bv.3
32	+/−	−	+	+	−	+	+	+	+	*B. melitensis* bv.3
33	+/−	−	+	+	−	+	+	+	+	*B. melitensis* bv.3
34	+/−	−	+	+	−	+	+	+	+	*B. melitensis* bv.3
35	+/−	−	+	+	−	+	+	+	+	*B. melitensis* bv.3
36	+/−	−	+	+	−	+	+	+	+	*B. melitensis* bv.3
37	+/−	−	+	+	−	+	+	+	+	*B. melitensis* bv.3
38	+/−	−	+	+	−	+	+	+	+	*B. melitensis* bv.3
39	+/−	−	+	+	−	+	+	+	+	*B. melitensis* bv.3
40	+/−	−	+	+	−	+	+	+	+	*B. melitensis* bv.3
41	+/−	−	+	+	−	+	+	+	+	*B. melitensis* bv.3
42	+/−	−	+	+	−	+	+	+	+	*B. melitensis* bv.3
43	+/−	−	+	+	−	+	+	+	−	*B. melitensis* bv.2
44	+/−	−	+	+	−	+	+	−	+	*B. melitensis* bv.1
45	+/−	−	+	+	−	+	+	−	+	*B. melitensis* bv.1
46	+/−	−	+	+	−	+	+	+	+	*B. melitensis* bv.3
47	+/−	−	+	+	−	+	+	+	+	*B. melitensis* bv.3
48	+/−	−	+	+	−	+	+	+	+	*B. elitensis* bv.3
49	+/−	−	+	+	−	+	+	+	+	*B. melitensis* bv.3
50	+/−	−	+	+	−	+	+	+	+	*B. melitensis* bv.3
51	+/−	−	+	+	−	+	+	+	+	*B. melitensis* bv.3
52	+/−	−	+	+	−	+	+	+	+	*B. melitensis* bv.3
53	+/−	−	+	+	−	+	+	−	+	*B. melitensis* bv.1
54	+/−	−	+	+	−−	+	+	−	+	*B. melitensis* bv.1
55	+/−	−	+	+	−	+	+	−	+	*B. melitensis* bv.1
56	+/−	−	+	+	−	+	+	+	+	*B. melitensis* bv.3
57	+/−	−	+	+	−	+	+	−	+	*B. melitensis* bv.1
58	+/−	−	+	+	−	+	+	+	+	*B. melitensis* bv.3
59	+/−	−	+	+	−	+	+	+	+	*B. melitensis* bv.3
60	+/−	−	+	+	−	+	+	+	+	*B. melitensis* bv.3
61	+/−	−	+	+	−	+	+	−	+	*B. melitensis* bv.1
62	+/−	−	+	+	−	+	+	−	+	*B. melitensis* bv.1
63	+/−	−	+	+	−	+	+	+	+	*B. melitensis* bv.3
64	+/−	−	+	+	−	+	+	+	+	*B. melitensis* bv.3
65	+/−	−	+	+	−	+	+	+	+	*B. melitensis* bv.3
66	+/−	−	+	+	−	+	+	+	+	*B. melitensis* bv.3
67	+/−	−	+	+	−	+	+	+	+	*B. melitensis* bv.3
68	+/−	−	+	+	−	+	+	+	+	*B. melitensis* bv.3
69	+/−	−	+	+	−	+	+	+	+	*B. melitensis* bv.3
70	+/−	−	+	+	−	+	+	+	+	*B. melitensis* bv.3
71	+/−	−	+	+	−	+	+	+	+	*B. melitensis* bv.3

### Development of a new multiplex PCR for the identification at genus and species level

**Figure 1:**
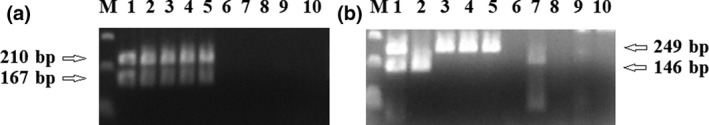
Identification of field endemic strains by multiplex PCR at genus (a) and species level (b). M: Marker (100bp), 1: *B. melitensis (Greek endemic strain),* 2: *B. abortus*, 3: *B. suis*, 4: *B. ovis*, 5: *B. canis*, 6: *Staphylococcus aureus*, 7: *Salmonella typhimurium*, 8: *Bacillus megaterium*, 9: *Shigella flexneri*, 10: *Escherichia coli 8879*.

Multiplex PCR consisted of two duplex PCRs. The first, based on the pairs of primers B4/B5 and BrucR/F (Fig. 1a), showed that the first five strains were of the genus *Brucella* as all gave products with both primer pairs, whereas the other five non‐related strains resulted in no PCR amplification products. The second duplex PCR, based on the pairs of primers JPF‐F/JPF‐abR and 1S‐F/1AS‐R, succeeded in identifying the strains at species level. As shown in Fig. 1b, none of the non‐related bacteria (Lanes 6–10) being used in the validation of the method gave the specific PCR products. Regarding the five *Brucella* species (Lanes 1–5), *B. melitensis* (Lane 1) was clearly identified as it gave bands of both 249 bp and 186 bp, *B. abortus* (Lane 2) was identified by giving a band only at 186 bp, whereas *B. suis, B. ovis* and *B. canis* (Lanes 3–5 respectively) gave the same pattern amplifying only the DNA fragment of 249 bp. After the standardization, the method was applied on all 71 endemic strains and identified them as *B. melitensis*. The analytical sensitivity of PCR was determined by seven serial dilutions (1:2, 1:5, 1:10, 1:20, 1:50, 1:100 and 1:500) of DNA as 0.1 ng for *B. melitensis* (data not shown).

### Identification at genus and species level by real‐time PCR analysis

**Figure 2:**
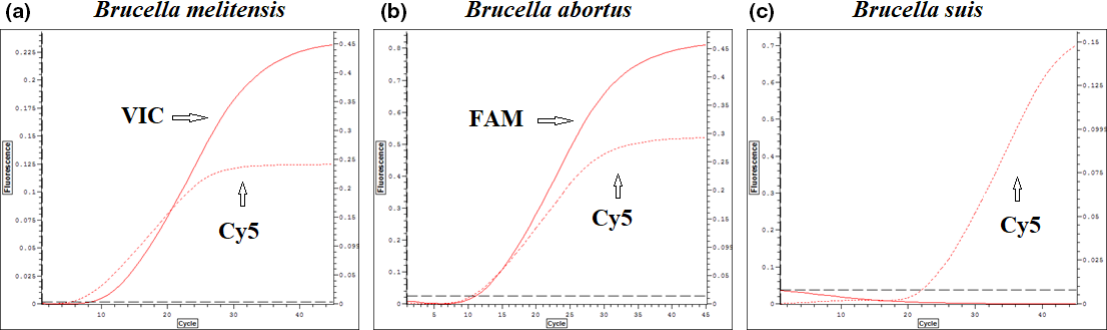
Identification of *Brucella* spp. field endemic strains by real‐time PCR. The probe Cy5 identifies the genus, while the probes FAM and VIC identify *B. abortus* and *B. melitensis* respectively.

For the real‐time PCR assay, two duplex qPCRs were performed on genomic DNA extracted from the samples' bacterial cultures. The efficiency of the real‐time PCR was estimated more than 98%. As shown in Fig. 2a, B*. melitensis* was identified by amplifying both genes, the multiple insertion element IS711 (probe Cy5 ‐ Ct: 5.24) and the BMEII0466 gene (probe VIC ‐ Ct: 8.39). Moreover, *B. abortus* was identified by amplifying the multiple insertion element IS711 (probe Cy5 ‐ Ct: 10.57) and the BruAb2_0168 gene (probe FAM ‐ Ct: 11.13) (Fig. 2b), whereas *B. suis* (Fig. 2c)*, B. ovis* and *B. canis* only amplified the multiple insertion element IS711, which is labelled with the probe Cy5 (Ct: 22.62) (probes FAM and VIC are non‐detected). The qPCR was also performed on the five pathogenic non‐related bacteria and resulted in no amplification products (data not shown). The analytical sensitivity of real‐time PCR was determined by serial dilutions of DNA as 0.1 ng for *B. melitensis* (data not shown). All Greek endemic strains in our study were clearly identified as *B. melitensis* as they amplified only the two genes labelled with the probes VIC and Cy5. The procedure of the two duplex qPCRs approved to be highly specific when applied on genomic DNA extracted after strain cultivation.

### Discrimination from vaccine strain Rev.1 by the analysis of omp2 gene by PCR‐RFLP

**Figure 3:**
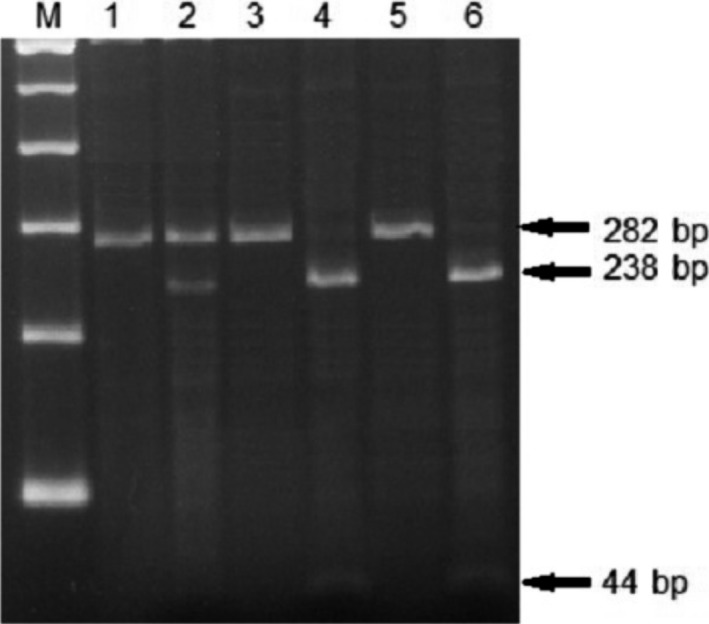
Discrimination of *Brucella melitensis* strains from vaccine strain Rev.1 by PCR‐RFLP assay. M: Marker (100 bp). Lane 1: Vaccine strain Rev.1 uncut. Lane 2: Vaccine strain Rev.1 digested with *Pst*I enzyme. Lanes 3 and 5: Uncut *B. melitensis* field strains of animal and human origin respectively. Lanes 4 and 6: Digested *B. melitensis* field strains of animal and human origin respectively.

As shown in Fig. 3, digestion of the amplified fragments of all field strains by *Pst*I restriction endonuclease revealed different bands on agarose gels compared with those of the vaccine strain. This is attributed to the fact that the *omp2* gene consists of two nearly homologous repeated copies, *omp2a* and *omp2b* alleles. Both *omp2* alleles (*omp2a* and *omp2b*) in the field strains have the *Pst*I recognition site, whereas the vaccine strain Rev.1 possesses the site only in the *omp2a* allele. Therefore, the digestion of the field endemic strains results in two fragments (−238 bp and −44 bp), while the digestion of the vaccine strain Rev.1 results in three fragments (−238 bp, −44 bp and the uncut fragment of the *omp2b* allele, −282 bp) (Noutsios *et al*. 2012).

## Discussion

In our study, a new multiplex PCRs have been developed to identify the *Brucella* genus and discriminate *B. melitensis* and *B. abortus* from three other species, namely *B. suis, B. ovis* and *B. canis*. Results obtained have shown that *B. melitensis* and *B. abortus* can be clearly differentiated from the other three *Brucella* species as well as from the non‐related pathogenic bacteria being used for the validation of the method's specificity. Moreover, the fact that the method allows the differentiation between *B. melitensis* and *B. ovis* could be useful in regions where sheep and goats are bred. The primers used in multiplex PCR protocol were highly specific for identifying *Brucella* at genus and species level, when the method was performed on genomic DNA after strain cultivation. The new method clearly identified all field endemic strains as *B. melitensis*. These findings were comparable to those obtained by real‐time PCR and microbiological methods, demonstrating that the developed multiplex PCR in our study is a rapid assay which could be implemented on routine brucellosis diagnosis, replacing reliably and effectively the techniques currently used.

Regarding the epidemiological aspects, there is a lack of data concerning the predominance of *B. melitensis* biovars in Greece. Previous studies being performed from 1977 to 1985 (Verger & Plommet 1985), identified eight out of 61 field strains as *B. melitensis* biotype 1 (13,11%) and 53 out of 61 strains as *B. melitensis* biotype 3 (86,89%). However, an unpublished research in 1996 identified all 16 isolated field strains as *B. melitensis* biotype 3. Since then, except for a brucellosis outbreak investigation in Thassos in 2008, where *B. melitensis* biotype 3 was identified in two clinical specimens (Karagiannis *et al*. 2012), there has been a lack of data concerning this species' biovar predominance. The bacteriological biotyping of *B. melitensis* strains being performed in our study for the interval 1999–2010, revealed that in Greece the predominant type is *B. melitensis* biovar 3 being slightly greater than *B. melitensis* biovar 1 These results are in accordance with the findings of other researchers (Samadi *et al*. 2010) who have demonstrated that *B. melitensis* biovar 3 is the most prevalent biovar in the Mediterranean and Middle East countries, whereas biotypes 1 and 2 are found mainly in southeastern Europe (Benkirane 2006). Moreover, our results showed that biotype 3 appeared in all the three regions of Greece examined between 1999 and 2010, in contrast to the other two biotypes (1 and 2) which were mainly isolated in mainland (northern and central) Greece. However, according to our findings and a previous study (Verger & Plommet 1985) biovar 2 has scarcely been isolated in Greece during the period 1977–2010, since only one strain was identified as *B. melitensis* biovar 2 in a region of northern Greece.

As systematic vaccination has been implemented in the mainland since 1975, it was considered essential to discriminate vaccine from field strains using PCR‐RFLP methodology. Other techniques being used for the differentiation of *B. melitensis* field strains from the vaccine strain Rev.1 include a multiplex PCR for all species of *Brucella* spp. (Garcia‐Yoldi *et al*. 2006) and a duplex PCR based on two primer pairs in one step (Alvarez *et al*. 2017). The PCR‐RFLP method performed in our study, confirmed that none of the 71 *B. melitensis* Greek endemic strains matched a vaccine strain. This result shows that all reported cases concerning the above isolates were caused by natural infection of herds and not due to vaccine strain horizontal spread, whereas at the same time it provides additional evidence that PCR‐RFLP assay can differentiate vaccine from field strains efficiently, timely, reliably and cost‐effectively. As brucellosis is recognized as an occupational disease, PCR‐RFLP method could be effectively used in discriminating field from vaccine strains not only in animals but also in humans who belong to the high risk occupational groups (veterinarians, abattoir workers, laboratory personnel).

In conclusion, the new multiplex PCR assay developed in our study is able to identify *Brucella* strains at genus and species level as the results compared with those obtained from both conventional and molecular techniques regarding *Brucella* spp. identification were equal and can all be used, PCR‐RFLP assay enabled us to reliably distinguish *B. melitensis* vaccine strain Rev.1 from Greek field strains and based on our findings the dominant type of *B. melitensis* in Greece during the period 1999–2010 is biotype 3.

Possible limitations of this study could be the relative small number of strains used. Further studies could possibly extend the monitoring period and sample load and also could focus on other identification and differentiation techniques for *Brucella* spp. in order to be quicker and of lower cost. MALDI‐TOF MS could be such an option, which is something already under research.

## Source of funding

This study was funded by the FP7 – EMIDA ERANET program “*Brucella melitensis*: biotyping and differential diagnostic ‐ Brucmel” and the States Scholarship Foundation, Greece.

## Conflict of interest

The authors declare that they have no conflict of interest.

## Ethics statement

The study protocol was approved with a waiver for informed consent by the Scientific Committee of the Postgraduate Program of Applied Public Health and Environmental Hygiene, Faculty of medicine, University of Thessaly, Larissa, Greece. The authors confirm that the ethical policies of the journal, as noted on the journal's author guidelines page, have been adhered to and the appropriate ethical review committee approval has been received. The US National Research Council's guidelines for the Care and Use of Laboratory Animals were followed. All procedures performed involving human participants were in accordance with the ethical standards of the institutional and/or national research committee and with the 1964 Helsinki declaration and its later amendments or comparable ethical standards.

## Notes

1 OXOID, Wade Road, Basingstoke, Hampshire, RG24 8PW, UK

2 Thermo Fisher Scientific, Invitrogen, Carlsbad, USA

3 Applied Biosystems Inc., 850 Lincoln Centre Drive Foster City, CA 94404, USA

4 Thermo Fisher Scientific, Invitrogen

5 Applied Biosystems Inc.

6 New England BioLabs^®^Ltd, 75‐77 Knowl Piece, Hitchin SG4 0TY, UK
